# Classical and non-classical HLA class I aberrations in primary cervical squamous- and adenocarcinomas and paired lymph node metastases

**DOI:** 10.1186/s40425-016-0184-3

**Published:** 2016-11-15

**Authors:** Debbie M. Ferns, A. Marijne Heeren, Sanne Samuels, Maaike C. G. Bleeker, Tanja D. de Gruijl, Gemma G. Kenter, Ekaterina S. Jordanova

**Affiliations:** 1 Center Gynecological Oncology Amsterdam (CGOA), Department of Obstetrics and Gynecology, VU University Medical Center, De Boelelaan 1117, 1081 HV Amsterdam, The Netherlands; 2 Center Gynecological Oncology Amsterdam (CGOA), Department of Gynecology, Netherlands Cancer Institute - Antoni van Leeuwenhoek, P.O. Box 90203, 1006 BE Amsterdam, The Netherlands; 3 Department of Pathology, VU University Medical Center, De Boelelaan 1117, 1081 HV Amsterdam, The Netherlands; 4 Department of Medical Oncology, VU University Medical Center-Cancer Center Amsterdam, De Boelelaan 1117, 1081 HV Amsterdam, The Netherlands; 5 Center Gynecological Oncology Amsterdam (CGOA), Department of Obstetrics and Gynecology, Amsterdam Medical Center, Meibergdreef 9, 1105 AZ Amsterdam, The Netherlands

**Keywords:** Cervical cancer, Primary tumor, Metastatic lymph nodes, Classical and non-classical HLA expression, Squamous cell carcinoma, Adenocarcinoma

## Abstract

**Background:**

Tumors avoid destruction by cytotoxic T cells (CTL) and natural killer (NK) cells by downregulation of classical human leukocyte antigens (HLA) and overexpression of non-classical HLA. This is the first study to investigate HLA expression in relation to histology (squamous cell carcinoma (SCC) vs. adenocarcinoma (AC)), clinicopathological parameters and survival in a large cervical cancer patient cohort.

**Methods:**

Classical (HLA-A and HLA-B/C)- and non-classical HLA molecules (HLA-E and HLA-G) were studied on primary tumors and paired lymph node (LN) metastases from cervical cancer patients (*n* = 136) by immunohistochemistry. The Chi^2^ test was used for the comparison of clinicopathological characteristics between SCC and AC patients. The Related-Samples Wilcoxon Signed Rank test was used to compare HLA expression between the primary tumor and metastasis in LN. Patient survival rates were analyzed by Kaplan-Meier curves and Log Rank test. The Mann-Whitney *U* Test was used to compare the distribution of HLA class I expression between SCC and AC.

**Results:**

Decreased expression of HLA-A (SCC *P* < 0.001), HLA-B/C (SCC *P* < 0.01; AC *P* < 0.01) and total classical HLA (SCC *P* < 0.001; AC *P* = 0.02) was apparent in metastatic tumor cells compared to the primary tumor. In primary SCC, there was a clear trend towards complete loss of HLA-A (*P* = 0.05). SCC metastases showed more complete loss of HLA-A, while AC metastases showed more complete loss of HLA-B/C (*P* = 0.04). In addition, tumor size and parametrium involvement were also related to aberrant HLA class I expression. No significant associations between HLA expression and disease-specific (DSS) or disease-free survival (DFS) were found in this advanced disease cohort. However, in the SCC group, samples showing loss of HLA-A or loss of total classical HLA but positive for HLA-G were linked to poor patient survival (DSS *P* = 0.001 and *P* = 0.01; DFS *P* = 0.003 and *P* = 0.01, for HLA-A and total classical HLA, respectively).

**Conclusion:**

These results strengthen the idea of tumor immune escape variants leading to metastasis. Moreover, SCC tumors showing downregulation of HLA-A or total classical HLA in combination with HLA-G expression had poor prognosis. Our findings warrant further analysis of HLA expression as a biomarker for patient selection for CTL- and NK- cell based immunotherapeutic intervention.

**Electronic supplementary material:**

The online version of this article (doi:10.1186/s40425-016-0184-3) contains supplementary material, which is available to authorized users.

## Background

A persistent human papilloma virus (HPV) infection and concomitant expression of the HPV E6 and E7 oncogenes are important mediators of the development of cervical cancer [1]. Principally, HPV-antigens and tumor-derived antigens should trigger activation of the immune system and subsequent destruction of infected cells and/or burgeoning (pre-) malignant lesions. However, HPV-infected and transformed cells can acquire a number of immune escape mechanisms to avoid the host’s immune system, resulting in eventual tumor growth and lymph node (LN) metastasis [2–4].

One of the mechanisms by which tumor cells can escape immune destruction, is downmodulaton of classical human leukocyte antigens (HLA) class I (HLA-A, -B, and -C) expression. These molecules are responsible for tumor-associated antigen presentation at the cell surface for recognition by cytotoxic T cells (CTLs) and targeted cell lysis [2–6]. In addition, tumor cells are able to increase the expression of non-classical HLA class I molecules (HLA-E and HLA-G), which can interact with the inhibitory receptors CD94/NKG2A and KIR2DL4/p49 on natural killer (NK) cells, as well as on effector T cells and myeloid cells (e.g., ILT2 and ILT4), leading to decreased NK cell and/or T cell effector activity and hereby potential tumor progression [7–11]. However, it has also been reported that KIR2DL4 can act as a stimulatory molecule [12]. In addition, HLA-E can also bind the stimulatory CD94/NKG2C receptor of NK cells, this might have less impact because of a 6-fold lower affinity as compared to the inhibitory receptors [13].

In cervical cancer, we and others have described loss of classical HLA class I [14–23], and expression of HLA-E and HLA-G at the site of the primary tumor [23–25]. In addition, some studies have compared HLA class I expression between primary cervical carcinoma and paired metastatic LNs [26–28]. In these studies, the patient groups were small and no comparison was made between the different histological subtypes, squamous cell carcinoma (SCC) and adenocarcinoma (AC), despite substantial differences between these two types regarding clinical outcome, oncogenic mutations, and immunological characteristics as shown by us and by others [24, 29–36].

The HLA class I status of primary and metastasized cervical tumor cells could be of clinical relevance to predict the response to chemotherapy [37] and to immunotherapy focusing on activating CTLs or NK cells in order to destroy tumor cells [38, 39]. In this study, we compared the expression of classical and non-classical HLA molecules between SCC and AC in primary tumors and synchronous metastatic LNs in a large group of patients, and linked these data with clinicopathological characteristics and outcome.

## Methods

### Patients and ethical approval

From 136 patients with cervical cancer and LN metastasis (SCC *n* = 103 and AC *n* = 33) diagnosed between 1986 and 2008, formalin-fixed, paraffin-embedded tissue blocks with primary cervical cancer and paired metastasis-positive LN were obtained from the archives of the Departments of Pathology at the VU University Medical Center (VUmc) Amsterdam (*n* = 42), Academic Medical Center (AMC) Amsterdam (*n* = 86), and Leiden University Medical Center (LUMC) (*n* = 8) in The Netherlands. The main clinicopathological characteristics of the patients were retrieved from the databases available at the Pathology departments and Gynecology departments at the different institutes and are summarized in Table 1. None of the patients underwent chemotherapy or radiotherapy before surgery.

**Table 1:** Clinicopathological characteristics of the study cohort

Clinicopathological characteristics	SCC	AC	*P* - value
Number of patients	103	33	–
Age			0.103^(#)^
Mean	44.25	41.39	
Min	24	23	
Max	81	72	
FIGO stage			0.856
IBI	60 (58.2)	20 (60.6)	
> = IBII	42 (40.8)	13 (39.4)	
Missing	1 (1)	0 (0.0)	
Tumor size			0.448
< =4 cm	72 (69.9)	25 (75.8)	
> 4 cm	29 (28.2)	7 (21.2)	
Missing	2 (1.9)	1 (3.0)	
Vaginal involvement			0.191*
Yes	17 (16.5)	1 (3.0)	
No	84 (81.6)	22 (66.7)	
Missing	2 (1.9)	10 (30.3)	
Parametrium invasion			0.122
Yes	38 (36.9)	7 (21.2)	
No	63 (61.2)	24 (72.7)	
Missing	2 (1.9)	2 (6.1)	
Progression			0.303
Yes	32 (31.1)	13 (39.4)	
No	65 (63.1)	14 (42.4)	
Missing	6 (5.8)	6 (18.2)	
Recurrence in 5 years			0.014
Yes	30 (29.1)	18 (54.5)	
No	64 (62.1)	14 (42.4)	
Missing	9 (8.7)	1 (3.0)	

Progression: determined by physical examination, pathological or radiological assessment

Recurrence: recurrence after a complete remission due to the treatment

*P*-value measured with Asymptotic Pearson Chi^2^; (*)Exact test was used when counts were less than 5; (^#^)Mann-Whitney *U* Test was used for mean comparison

Data shown as n (%)

*FIGO* International Federation of Gynecology and Obstetrics, *SCC* squamous cell carcinoma, *AC* adenocarcinoma

All human tissue samples in this study were coded anonymous, and were used according to the medical ethical guidelines described in the Code Proper Secondary Use of Human Tissue established by the Dutch Federation of Medical Sciences (http://www.federa.org) [40].

### Immunohistochemistry

Formalin-fixed, paraffin-embedded tissue blocks were sectioned at 4 μm and mounted on StarFrost slides (Waldemar Knittel, Germany). Slides were deparaffinized in 3× xylene and washed in 1× 100 %, 1× 90 % of ethanol. Then, endogenous peroxidase was blocked with 0.3 % H_2_O_2_ (MERCK, Germany) in methanol for 20 min. Slides were rehydrated in 1× 70 % of ethanol and 1× demineralized water and heated in a microwave for antigen retrieval for 10 min in boiling 0.01 M citrate buffer pH 6.0 (for classical HLA-A, -B/C and HLA-E) or Tris/EDTA buffer pH 9.0 (for HLA-G). The slides were allowed to cool down for 1 h at room temperature (RT). After antigen retrieval, all slides were washed with 2× demineralized water and 2× phosphate buffered saline (PBS) and incubated over night at RT with one of the following antibodies diluted in 1 % BSA/PBS; mouse-anti-HCA2 (HLA-A), mouse-anti-HC10 (HLA-B/C) (both antibodies provided by Prof. Neefjes from the Dutch Cancer Institute, NKI-AvL, Amsterdam), mouse-anti-HLA-E (MEM-E/02; AbD Serotec, UK), and mouse-anti-HLA-G (4H84; BD Pharmingen™, USA). The next day, slides were washed 3× in PBS and incubated with BrightVision (ImmunoLogic, The Netherlands) for 30 min at RT. Then, slides were washed 3× in PBS, after which immune complexes were visualized with 0.05 % solution of 3,3′-Diaminobenzidine (DAB) containing 0.0018 % H_2_O_2_ in a 0.05 M Tris–HCl buffer (pH 7.6) for 10 min in the dark at RT. Finally, the sections were counterstained with Haematoxylin followed by 5 min rinsing with running tap water. Finally, slides were dehydrated and mounted under coverslips with Quick-D mounting medium (Klinipath, The Netherlands).

### Imaging and scoring

Sections were scored by the percentage and intensity of the immunostained tumor cells using an Olympus BX50 bright-field microscope (Olympus, USA) by three investigators (D.M.F., A.M.H., and E.S.J.). Stromal cells and infiltrating immune cells served as an internal positive control for HLA-A, -B/C and -G detection, while vascular cells served as an internal positive control for HLA-E detection. The percentage of classical HLA class I positive tumor cells was scored as 0 for absent, 1 for sporadic (1–5 %), 2 for local (6–25 %), 3 for occasional (26–50 %), 4 for majority (51–75 %), and, 5 for large majority (76–100 %). Secondly, the intensity of the staining was scored as 0 (absent), 1 (dull), 2 (clear), or 3 (strong), based on comparison with the normal cells present in the same sample. The sum of both scores (percentage and intensity) were used to identify three categories for classical HLAs (0–2 as complete loss of expression, 3–6 as weak expression, and 7–8 as normal expression) and two categories for non-classical HLAs (0–4 as no expression and 5–8 as expression) as previously described by Ruiter et al. [41]. For non-classical HLA score a cut off of 5 was used to define groups [24]. Total classical HLA scoring was based on the combined scores of HLA-A and -B/C [21].

### Statistical analysis

All statistical analyses were performed using SPSS 20 statistical software (SPSS 20.0, SPSS Inc. Chicago, IL, USA). The Chi^2^ tests (Pearson Chi^2^ and Linear-by-Linear Association, Asymptotic or Exact tests, two-sided) were used for the comparison of clinicopathological characteristics between SCC and AC. The Related-Samples Wilcoxon Signed Rank test was used to compare HLA expression between the primary tumor and metastasis in LN. Patient survival rates were analyzed by Kaplan-Meier curves and the Log Rank (Mantel-Cox) test. The Mann-Whitney *U* Test was used to compare the distribution of HLA class I expression between SCC and AC. Differences were considered statistically significant when *P* < 0.05.

## Results

### Clinicopathological characteristics of SCC vs AC

**Fig. 1:**
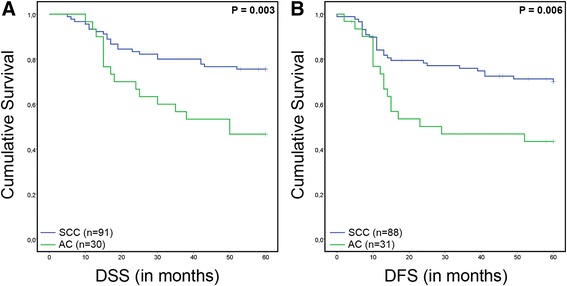
Survival analysis stratified for SCC and AC subtypes. 5-year Kaplan Meier survival curves and Log Rank test show a significantly poorer disease specific survival (DSS) (**a**) and disease free survival (DFS) (**b**) for patients with metastatic AC compared to patients with metastatic SCC. For SCC, DSS data was missing for 12 and DFS data- for 15 cases. For AC, DSS data was missing for 3 cases and DFS data for 2 cases

Patients diagnosed with metastatic AC, manifested with a higher recurrence rate (*P* = 0.014, Chi^2^ test) (Table 1). Furthermore, these patients had a worse 5-year disease-specific (DSS) and disease-free survival (DFS) compared to patients with SCC (*P* = 0.003 and *P* = 0.006, respectively, Log Rank test) (Fig. 1).

### HLA class I expression in primary cervical cancer and paired metastatic LN

**Fig. 2:**
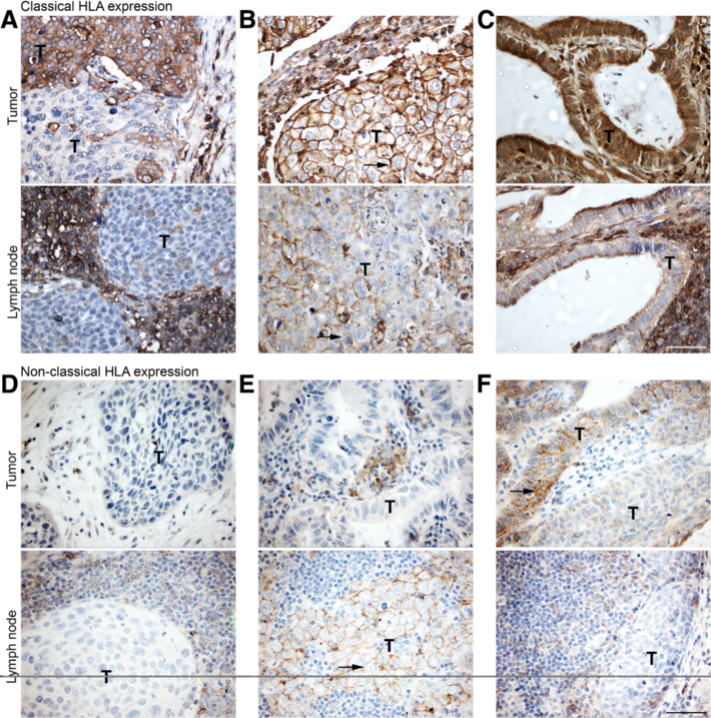
Representative examples of HLA expression patterns in the primary tumor and corresponding metastatic tumor sample. **a** Heterogeneous HLA-A expression in SCC on the cell membrane of the primary tumor cells (T). In the corresponding LN, no HLA-A was expressed by the metastatic tumor cells (T in lower panel), while it is expressed by surrounding immune cells. **b** Primary tumor cells (T) show membrane expression of HLA-A (indicated by black arrow, upper panel). Metastatic tumor cells (T in lower panel) in corresponding LN show less expression of HLA-A compared to the primary tumor. **c** Primary AC shows a high cytoplasmic expression of HLA-A (T in upper panel). In the corresponding LN metastasis, there is loss of HLA-A expression in the tumor cells (T in lower panel), while it is expressed by surrounding immune cells. **d** Low expression levels of HLA-G in primary SCC (T in upper panel) and in the corresponding metastatic tumor cells (T in lower panel). **e** Primary AC (T in upper panel) show low expression levels of HLA-G, while in the corresponding metastatic tumor cells (T in lower panel), HLA-G is abundantly expressed at the cell surface. **f** Primary AC (T in upper panel) show high expression levels of HLA-G, while HLA-G is not expressed in the corresponding metastatic tumor cells (T in lower panel). Scale bar is 50 μm

**Fig. 3:**
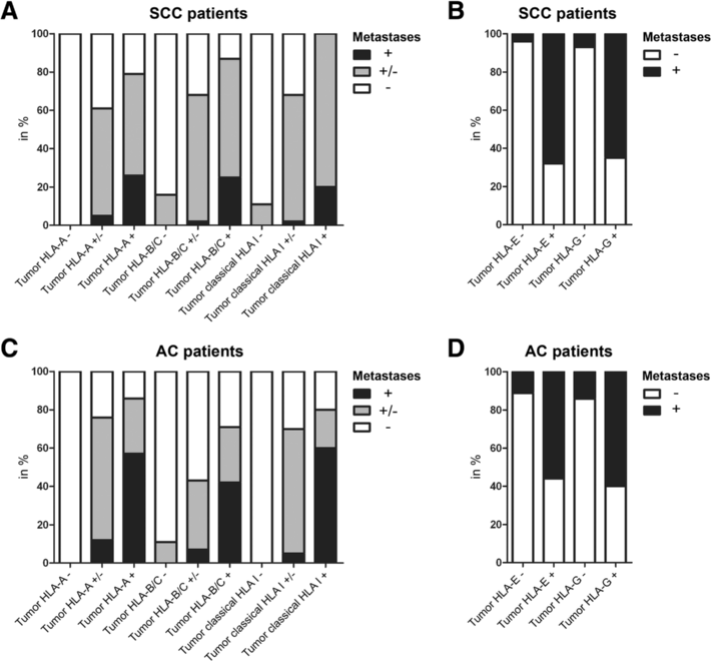
HLA expression distribution in primary tumor samples compared to metastatic tumor samples. Classical (**a**) and non-classical (**b**) HLA class I expression of primary tumor samples compared to metastatic tumor samples of patients with SCC. Classical (**c**) and non-classical (**d**) HLA class I expression of primary tumor samples compared to metastatic tumor samples of patients with AC. In A and C: ‘+’ is normal expression, ‘+/-‘is weak expression, and ‘-‘is complete loss. In B and D: ‘-‘is no expression and ‘+’ is normal expression

HLA class I expression in paired primary tumor and LN metastasis samples of SCC and AC was analyzed by immunohistochemistry for HLA-A, HLA-B/C, HLA-E and HLA-G. Representative examples of classical and non-classical HLA expression are depicted in Fig. 2. The results obtained for HLA class I expression are summarized in Additional file 1: Table S1 and shown in Fig. 3.

#### Classical and non-classical HLA class I expression in SCC

Most primary SCC tumors manifested with downregulation (including cases both with complete loss and weak expression) of HLA-A (79 %), HLA-B/C (90 %), and total classical HLA expression (94 %). Metastatic tumor cells in the LN, showed also downregulation of HLA-A (92 %), HLA-B/C (96 %), and total classical HLA (98 %). Unsurprisingly, there was a clear association between loss of HLA in the primary tumor and the metastatic LN (Additional file 1: Table S1). However, more importantly, we found a significantly lower expression of classical HLA class I in SCC paired metastatic tumor cells for HLA-A (*P* < 0.001, Wilcoxon Signed Rank test), HLA-B/C (*P* = 0.005, Wilcoxon Signed Rank test), and total classical HLA (*P* < 0.001, Wilcoxon Signed Rank test).

In addition, HLA-E expression was detected in 37 % and HLA-G- in 22 % of the primary tumors (Additional file 1: Table S1). There was significantly less HLA-E expression in the metastatic LN than in the paired primary tumor (*P* = 0.035, Wilcoxon Signed Rank test). No other significant correlations were found.

#### Classical and non-classical HLA class I expression in AC

In AC, most tumors manifested with downregulation (including cases both with complete loss and weak expression) of HLA-A (77 %), HLA-B/C (77 %), and total classical HLA (84 %). At the metastatic LN, tumor cells also showed downregulation of HLA-A (80 %), HLA-B/C (87 %), and total classical HLA (87 %). As was the case for SCC, in AC there was a clear association between loss of HLA in the primary tumor and the metastatic LN (Additional file 1: Table S1). In the paired metastatic AC samples a significantly lower expression of HLA-B/C (*P* = 0.007, Wilcoxon Signed Rank test) and total classical HLA (*P* = 0.021, Wilcoxon Signed Rank test) was found compared to the primary tumor.

At the site of the primary tumor, we observed 33 and 31 % expression of HLA-E and HLA-G, respectively. The metastatic tumor cells showed less expression of HLA-E (26 %) and HLA-G (28 %), however, this was not significant.

#### Comparison of HLA class I expression between SCC and AC

**Fig. 4:**
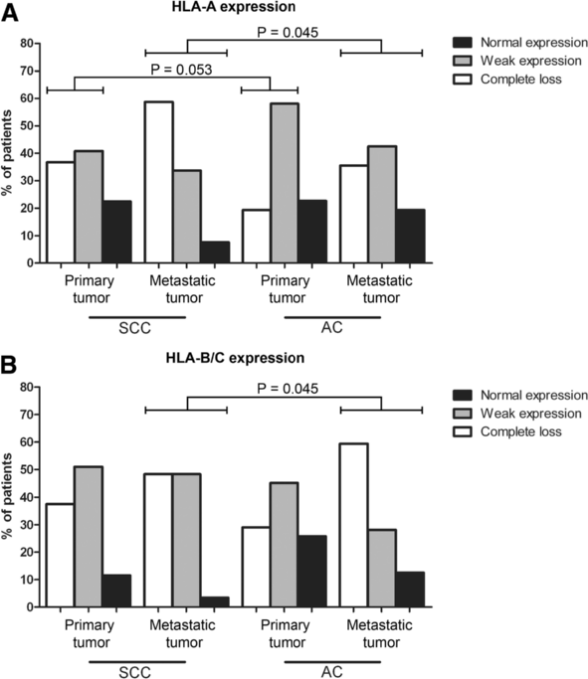
Comparison of HLA expression patterns in primary tumor and metastatic tumor samples. The distribution of (**a**) HLA-A and (**b**) HLA-B/C expression in LN is significantly different between patients with SCC and AC (for both *P* = 0.045, Chi^2^ test). In SCC, more complete loss of HLA-A expression was observed in primary tumor and LN, while in AC, there was more weak expression of HLA-A in primary (*P* = 0.053, pairwise Chi^2^ test) tumor and LN (*P* = 0.081, pairwise Chi^2^ test). NB: We cannot exclude the possibility of HLA haplotype loss or HLA allelic loss as previously described [19] in the cases defined as having ‘normal HLA expression’ since these were solely based on HCA2 and HC10 staining

When complete loss and weak expression of HLA-A were compared between the histological subtypes, we found a trend toward more complete loss in primary SCC (*P* = 0.053, Chi^2^ test - pairwise) and SCC metastatic LN (*P* = 0.081, Chi^2^ test - pairwise) (Fig. 4a). In the metastatic LN, HLA-A and HLA-B/C expression patterns were significantly different between SCC and AC (for both *P* = 0.045, Chi^2^ test); in SCC, there were more LNs with complete loss of HLA-A expression (Fig. 4a), while in AC there were more LNs with complete loss of HLA-B/C (Fig. 4b). No significant differences for the remaining HLA molecules were found between SCC and AC for the primary tumor or the LN.

### Association of HLA class I expression and clinicopathological parameters

In SCC, larger tumors (= > 40 mm) showed more often complete loss as compared to partial loss of HLA-A (40 % vs. 17 %) and total classical HLA (45 % vs. 21 %) expression (*P* = 0.034 and *P* = 0.027, Chi^2^ test – pairwise). In addition, parametrium involvement was associated with complete as compared to partial loss of HLA-B/C (54 % vs. 31 %) and total classical HLA (54 % vs. 33 %) in the metastatic tumor cells (*P* = 0.037 and *P* = 0.050, Chi^2^ test – pairwise). Surprisingly, expression of HLA-G (in 83 % of the cases) in the metastatic tumor cells was also significantly more often present in cases without parametrium involvement (*P* = 0.034, Chi^2^ test).

In AC, larger tumors (= > 40 mm) showed more often complete loss (67 %) compared to partial loss (14 %) of HLA-A expression (*P* = 0.037, Chi^2^ test - pairwise). No other significant associations were found.

### Survival and HLA class I expression

Log rank test was performed and Kaplan-Meier plots were generated for DSS and DFS to assess the correlation with classical and non-classical HLA expression in the primary tumor and metastatic LN. We found no significant associations between classical HLA class I expression and DSS (Additional file 2: Figure S1) nor DFS (not shown) for both histological types. Noticeably, patients with cervical AC with weak HLA-A expression in the primary tumor cells had the poorest DSS (not significant). Furthermore, AC patients with HLA-E expression in the primary tumor also appeared to have a poor DSS (not significant).

**Fig. 5:**
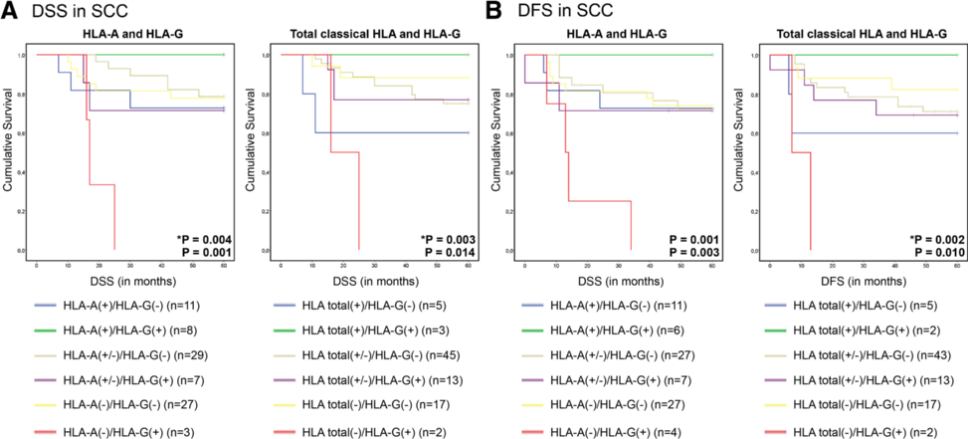
Survival plots for SCC patients, combining classical HLA and HLA-G expression in the primary tumor. 5 year disease-specific survival (DSS) (**a**) and disease-free survival (DFS) (**b**) curves for SCC patients in relation to HLA-A/HLA-G expression and total classical HLA/HLA-G expression in the primary tumor. Both patient groups with down-regulated HLA-A(-) or total classical HLA(-) in combination with HLA-G expression had the poorest survival (*red line*). Log Rank test *P*-value combining all groups, * Log Rank test *P*-value between classical HLA(-)/HLA-G(+) (*red line*) and classical HLA(-)/HLA-G(-) (*yellow line*)

In addition, we analyzed combinations of classical and non-classical HLA expression patterns. In primary SCC two significant findings were made; patients without HLA-A expression but with HLA-G expression had the worst DSS (*P* = 0.001) and DFS (*P* = 0.003) (Fig. 5). The same was true for the patients with complete total classical HLA loss but with HLA-G expression (DSS *P* = 0.014, DFS *P* = 0.010) (Fig. 5).

Concerning DSS and DFS of AC patients, no significant correlations were observed and no conclusions could be drawn as the number of AC patients per group was too limited.

## Discussion

We believe the present study to be the first to include a large cervical cancer patient cohort and to report on the differences between SCC and AC, regarding classical and non-classical HLA expression in paired primary tumors and metastatic LN and to relate the expression patterns to clinicopathological characteristics and patient survival. In the past, only one publication on a small cervical cancer cohort (*n* = 26) has reported analysis on primary tumor and paired metastatic LN and has taken the two major histological subtypes into account [42].

Here, the vast majority of SCC and AC manifested with downregulation of classical HLA at the site of the primary tumor and even lower expression in the metastatic tumor cells. This phenomenon has been also observed in other tumor types, like breast-, lung-, and liver carcinomas [43, 44]. This is likely resulting in a decreased sensitivity to T cell lysis, which is supported by the observed significant correlation between HLA class I downregulation and a decrease in tumor-infiltrating CD8^+^ T cells [27, 45], with particularly lower numbers of CD8^+^ T cells in primary tumors with weak HLA-A expression [21]. Moreover, this outcome fits with the concept that tumor cells are positively selected based on low- or no expression of classical HLA, caused by genetic alterations like HLA class I allele-specific mutations, β2-microglobulin gene mutations, and antigen processing machinery-associated mutations [19, 22, 46, 47], and can be linked to invasiveness and metastatic potential [16, 48–50]. Previously, Menon et al. reported on the lack of downregulation of HLA class I in liver metastasis compared to primary colorectal cancer samples [51], however, we and others showed the opposite, probably explained by differences in the microenvironment of the metastatic site; liver metastasis versus lymph node metastasis. Interestingly, we did find a trend towards more HLA-A complete loss in primary SCC tumors compared to primary AC tumors, which was also observed in the LNs and supported by another study in cervical cancer patients [42]. In addition, complete loss of HLA-A was associated with larger tumors in both SCC and AC, which is consistent with a study in breast cancer reporting that larger tumors manifest with low HLA class I expression [44].

The crucial role of the non-classical HLA molecules was recently demonstrated in large cohorts of breast [52], ovarian [53] and colorectal [54] cancer patients. Until now only HLA-E was studied in SCC and AC primary cervical cancer samples and high expression of HLA-E was found in 56 %, with significantly higher expression in cervical AC compared to SCC [24]. In contrast, in the present study, we found 30 % expression of HLA-E in the primary tumor for both groups. This difference might be explained by the fact that we analyzed an advanced-stage, metastatic patient group, while HLA-E expression might be an earlier event in tumor progression [44]. In fact, in SCC, there was significantly less HLA-E expression in the metastatic tumor cells compared to the primary tumor.

In previous studies, HLA-G expression was found in 27–30 % of the primary- [28, 55] and 11 % of the metastatic tumor samples [28], but without histological subtyping. Here, comparable results were obtained with approximately 25 % of the cases having expression of HLA-G in the primary tumor and 11 % in the metastatic LNs. No significant differences were observed between histological subtypes.

Next to the association between primary tumor, metastases and clinical parameters, we analyzed the importance of classical HLA loss and non-classical HLA expression for patient survival. As here we have focused on an advanced, metastatic patient cohort these patients already had a worse prognosis than patients without metastases [56, 57]. We could identify significantly higher progression rates and a significantly poorer outcome for patients with metastatic AC compared to patients with metastatic SCC, which is in accordance with earlier studies showing AC to be the more aggressive subtype [58, 59]. Studies on penile cancer [60], colorectal cancer [61], and primary cervical cancer (including a mixed SCC and AC patient cohort with or without metastasis) [21], described a strong association between partial loss of HLA expression and poor prognosis. In contrast, we did not find significant associations between classical HLA expression and survival rates. Possibly, due to the obvious poor survival of this metastatic patient group, contrary to previous findings, we could not confirm survival advantage for AC patients with HLA-E expression in the primary tumor [24].

Although very low numbers of NK cells are present in cervical tumors [21, 62], downregulation of classical HLA may lead to decreased sensitivity of T cell lysis, but simultaneously may enhance sensitivity to NK cell lysis [48], we studied classical HLA expression in combination with non-classical HLA expression. Interestingly, SCC patients with HLA-G expression in combination with downregulation of HLA-A or total classical HLA in the primary tumor had a significantly poorer DSS and DFS, which is in accordance with a study in breast cancer [52]. Previous studies, including cervical cancer, also reported on HLA-G upregulation in case of classical HLA loss [44, 55, 63]. However, our findings should be interpreted with caution, because of the limited number of patients per group and the limited number of patients with normal expression of classical HLA in the present patient group (eight for SCC and five for AC). The same holds true for the metastases group; there were only two SCC metastases and four AC metastases with normal HLA class I expression.

## Conclusion

In conclusion, in both histological tumor types, SCC and AC, our results show evidence of aberrant patterns of HLA expression with even lower classical HLA class I expression in corresponding LN metastases. Our study is likely to be an under-representation of the actual HLA aberrations due to specific allele mutations, which cannot be identified by immunohistochemistry. In patients with primary SCC, complete loss of classical HLA was found more frequently than in patients with AC (*P* = 0.05), suggesting that this is one of the important mechanisms for tumor progression in SCC. Based on these findings, we speculate that there could be a more pronounced immunological pressure on SCC cells to completely downregulate classical HLA expression thereby becoming less sensitive for T cell lysis. Moreover, this immunological pressure is prominent in SCC tumors, in which downregulation of HLA-A or total classical HLA in combination with HLA-G expression is related to poor prognosis, suggesting escape from both T cell and NK cell antitumor activity.

It would be of great interest to study the differences in quality and quantity of HPV-specific immune responses between SCC and AC, since AC is more frequently associated with HPV18 [64]. Recently, Safaeian et al. found an association between HLA allelic sub-types and HPV type-specific peptides, suggesting that HLA recognition is HPV type-specific [65]. In addition, reduced levels of TNFα and IFNγ were found in HPV18-infected patients compared to HPV16-infected patients [66], and less IL-6 and IL-12 was present in the supernatant of the SiHa (HPV16+) cell line compared to the supernatant of the HeLa (HPV18+) cell line, while both cell lines were able to induce immunosuppressive M2 type marker CD163 expression on macrophages [67]. However, still not much is known about the immunological differences between SCC and AC. It is puzzling that AC retain higher HLA class I expression levels as compared to SCC. We have recently published on substantial differences between the SCC and AC histological subtypes regarding oncogenic mutations, with e.g., KRAS mutation exclusively found in AC [31]. In contrast, we found EGFR upregulation exclusively in SCC [68]. Currently, we are focusing on understanding the immunological differences between SCC and AC, and we are investigating the presence of different immune cell subsets which may give us more information on the different role of HLA expression in these histological subtypes [32–36]. In addition, there was more complete HLA-A loss in SCC metastasis, while there was more complete loss of HLA-B/C in AC metastasis, again emphasizing the need for better immune-characterization of the two major cervical cancer histological subtypes.

The translational relevance of these findings is potentially high with the exponential rise of T cell based immunotherapeutic approaches in the past few years [69, 70] as it is conceivable that tumor cells with low or absent classical HLA will not respond, or will respond differently, to these therapies due to reversible or irreversible HLA class I alterations [49, 71]. It will be of great interest to dissect the effect of classical and non-classical HLA expression in (metastatic) cervical cancer on the clinical effect of therapeutic vaccination and other immunotherapies, which will potentially lead to the selection of a patient group that is most likely to respond to this type of intervention.

## Additional files

Additional file 1: Table S1. HLA Expression pattern comparison between primary tumor and metastatic tumor samples. (DOC 56 kb)
Additional file 2: Figure S1. Survival plots of HLA expression in the primary tumor and paired metastatic tumor samples. 5-years disease specific survival (DSS) rates for patients with cervical SCC (A) and AC (B) in relation to HLA-A, HLA-B/C, total classical HLA, HLA-E and HLA-G expression in primary tumor cells and paired metastases. P-value was calculated by Log rank test. (TIF 20952 kb)

## Abbreviations

AC: Adenocarcinoma
CTLs: Cytotoxic T cells
DFS: Disease-free survival
DSS: Disease-specific survival
HLA: Human leukocyte antigens
HPV: Human papilloma virus
LN: Lymph node
SCC: Squamous cell carcinoma
