# Supplementary material for: Classical and non-classical HLA class I aberrations in primary cervical squamous- and adenocarcinomas and paired lymph node metastases
Source: J Immunother Cancer. 2016 Nov 15;4:78. doi: 10.1186/s40425-016-0184-3 (PMC5109766; doi:10.1186/s40425-016-0184-3)
Supplement: Additional file 1: Table S1. — HLA Expression pattern comparison between primary tumor and metastatic tumor samples. (DOC 56 kb) [file 40425_2016_184_MOESM1_ESM.doc]

**Additional file 1: Table S1: HLA expression pattern comparison between primary tumor and metastatic tumor samples.**

|  |  | | **Lymph node metastasis** | | | | |  |
| --- | --- | --- | --- | --- | --- | --- | --- | --- |
|  |  |  | **Complete loss** | | **Weak**  **expression** | **Normal**  **expression**** | | **P-value** |
| **Primary SCC** | **HLA-A** | **Complete loss** | 34 (100.0) | 0 (0.0) | | | 0 (0.0) |  |
|  |  | **Weak expression** | 14 (39.0) | 20 (55.5) | | | 2 (5.5) | < 0.001 |
|  |  | **Normal expression** | 4 (21.1) | 10 (52.6) | | | 5 (26.3) |  |
|  | **HLA-B/C** | **Complete loss** | 26 (83.9) | 5 (16.1) | | | 0 (0.0) |  |
|  |  | **Weak expression** | 14 (31.8) | 29 (65.9) | | | 1 (2.3) | < 0.001 |
|  |  | **Normal expression** | 1 (12.5) | 5 (62.5) | | | 2 (25.0) |  |
|  | **Total classical HLA*** | **Complete loss** | 16 (88.9) | 2 (11.1) | | | 0 (0.0) |  |
|  |  | **Weak expression** | 20 (32.3) | 41 (66.1) | | | 1 (1.6) | < 0.001 |
|  |  | **Normal expression** | 0 (0.0) | 4 (80.0) | | | 1 (20.0) |  |
|  |  | | **No expression** | **Normal**  **Expression** | | |  |  |
|  | **HLA-E** | **No expression Expression** | 45 (95.7)  9 (32.1) | 2 (4.3)  19 (67.9) | | |  | < 0.001 |
|  | **HLA-G** | **No expression Expression** | 65 (92.9)  7 (35.0) | 5 (7.1)  13 (65.0) | | |  | < 0.001 |
|  |  | | **Complete loss** | **Weak expression** | | | **Normal**  **expression**** |  |
| **Primary AC** | **HLA-A** | **Complete loss** | 6 (100.0) | 0 (0.0) | | | 0 (0.0) |  |
|  |  | **Weak expression** | 4 (23.5) | 11 (64.7) | | | 2 (11.8) | < 0.001 |
|  |  | **Normal expression** | 1 (14.3) | 2 (28.6) | | | 4 (57.1) |  |
|  | **HLA-B/C** | **Complete loss** | 8 (88.9) | 1 (11.1) | | | 0 (0.0) |  |
|  |  | **Weak expression** | 8 (57.1) | 5 (35.7) | | | 1 (7.2) | 0.004 |
|  |  | **Normal expression** | 2 (28.6) | 2 (28.6) | | | 3 (42.8) |  |
|  | **Total classical HLA*** | **Complete loss** | 6 (100.0) | 0 (0.0) | | | 0 (0.0) |  |
|  |  | **Weak expression** | 6 (30.0) | 13 (65.0) | | | 1 (5.0) | < 0.001 |
|  |  | **Normal expression** | 1 (20.0) | 1 (20.0) | | | 3 (60.0) |  |
|  |  | | **No expression** | **Normal Expression** | | |  |  |
|  | **HLA-E** | **No expression Expression** | 16 (88.9)  4 (44.4) | 2 (11.1)  5 (55.6) | | |  | 0.023 |
|  | **HLA-G** | **No expression Expression** | 19 (86.4)  4 (40.0) | 3 (13.6)  6 (60.0) | | |  | 0.013 |
