# Supplementary figures and images for: Classical and non-classical HLA class I aberrations in primary cervical squamous- and adenocarcinomas and paired lymph node metastases
Source: J Immunother Cancer. 2016 Nov 15;4:78. doi: 10.1186/s40425-016-0184-3 (PMC5109766; doi:10.1186/s40425-016-0184-3)

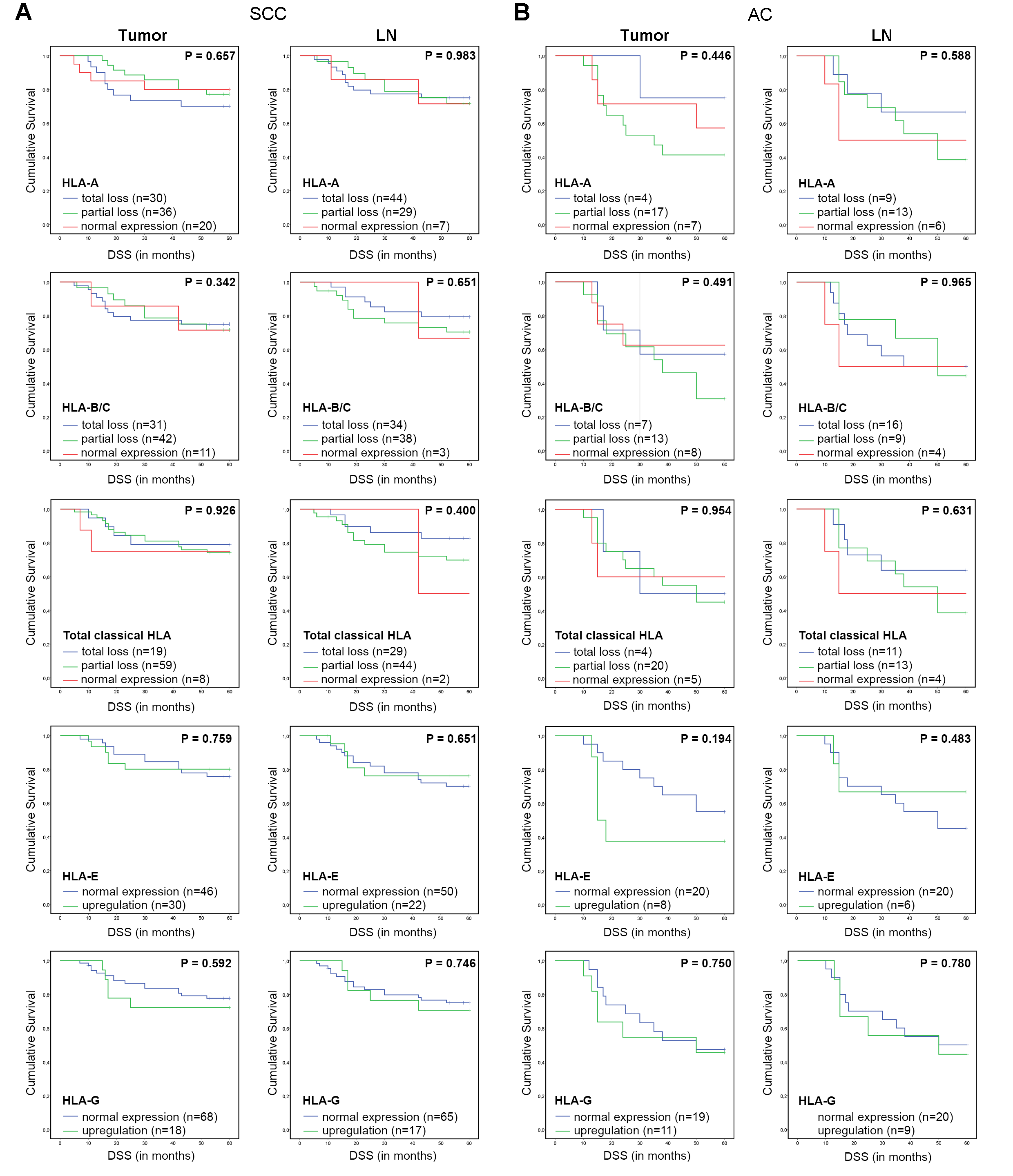

Supplement: Additional file 2: Figure S1. — Survival plots of HLA expression in the primary tumor and paired metastatic tumor samples. 5-years disease specific survival (DSS) rates for patients with cervical SCC (A) and AC (B) in relation to HLA-A, HLA-B/C, total classical HLA, HLA-E and HLA-G expression in primary tumor cells and paired metastases. P-value was calculated by Log rank test. (TIF 20952 kb) [file 40425_2016_184_MOESM2_ESM.tif]
